# Knowledge, attitudes, practices about HIV and implications in risk and stigma prevention among French Guianese and Brazilian border inhabitants: *Beliefs about HIV among border inhabitants*

**DOI:** 10.1186/s12889-019-7997-1

**Published:** 2019-12-04

**Authors:** E. Mosnier, M. Nacher, M. C. Parriault, C. Dao, B. Bidaud, P. Brousse, M. Gaillet, L. Epelboin, A. M. Mendes, L. Montenegro, C. Nakano Daniel, R. Botreau, A. Rouseliere, S. Rhodes, A. Carbunar

**Affiliations:** 1 Pôle des Centres Délocalisés de Prévention et de Soins, Centre Hospitalier Andrée Rosemon, Cayenne, French Guiana; 2 Aix Marseille University, INSERM, IRD, SESSTIM, Sciences Economiques & Sociales de la Santé & Traitement de l’Information Médicale, Marseille, France; 3 Centre d’Investigation Clinique Antilles Guyane, CIC INSERM 1424, Centre Hospitalier Andrée Rosemon, Cayenne, French Guiana; 4 Ecosystèmes Amazoniens et Pathologie Tropicale, EA3593, Université de Guyane, Cayenne, French Guiana; 5 Dsanté NGO, Rémire Montjoly, Rémire Montjoly, French Guiana; 6 Unité de Maladies Infectieuses et Tropicales, Centre Hospitalier Andrée Rosemon, Cayenne, French Guiana; 7 Universidade Federal do Amapá (UNIFAP), Oiapoque, Brazil

**Keywords:** Border, HIV, Sexual risk, Health knowledge attitudes behaviors practices, French Guiana, Brazil

## Abstract

**Background:**

The border area between French Guiana and Brazil is an active HIV-transmission zone. The aim of the present study was to describe HIV knowledge, risk and the level of stigma among inhabitants of this border area.

**Methods:**

A cross-sectional study was conducted among 621 inhabitants over 18 years of age in the border cities of Saint-Georges-de-l’Oyapock in French Guiana and Oiapoque in Brazil. It was conducted between October 2017 and February 2018. An anonymous standardized questionnaire was filled out by culturally-trained mediators, then analyzed using STATA 12.

**Results:**

Almost half (45.9%) of the individuals had a low education level. Participants whose native language was Portuguese or French demonstrated better HIV knowledge than other populations, notably native Amerindian and creole-speaking people. HIV risk behavior was more frequent in men and in younger age groups. People with good HIV knowledge reported having performed more HIV tests in the last year than participants with poor knowledge. The stigma level was high and reported in 74.8% of respondents.

**Conclusions:**

These results illustrate the need for initiatives to improve HIV prevention among autochthonous populations on both sides of this border area. Cross-border collaboration on health policies could produce common key messages adapted to the education level and multi-linguistic populations who live in this area.

## Background

French Guiana is a French overseas territory located in the northern region of Brazil. It is bordered by the State of Amapá. For more than 10 years, the HIV prevalence rates among pregnant women in French Guiana and in Amapá have exceeded 1% [1, 2]. In French Guiana and Brazil, guidelines are based on the “test and treat” program and are free of charge [3]. However, there are vulnerable key populations, notably among immigrants and border area inhabitants, where the epidemic remains active [3–6].

The border between French Guiana and Brazil is a corridor for immigrants but also a supply area for illegal gold miners called “Garimpeiros”, who come mainly from northern Brazil [7]. Sex tourism thrives in the area and high risk practices associated with HIV have been previously described among sex workers [8]. The border areas in French Guiana are precarious and frequent stigmatizing attitudes have been reported [9].

In French Guiana, many foreign HIV patients acquire the infection after entering this French territory [4]. HIV patients in French Guiana coming from Brazil and Surinam had a longer interval between seroconversion and diagnosis than patients with other nationalities [10]. Brazilian HIV patients are also more likely to receive follow-up care in small remote health care centers along the border than in urban hospital centers [6]. However, the border between French Guiana and the Brazilian State of Amapá is also an area where new strategies in terms of prevention, diagnosis and care are now being implemented to address these unique challenges. For example, strategies include pair-education, community training, PrEP and cross-border cooperation in caring for HIV patients [5]. Although assessments have been conducted among sex workers, there has never been any evaluation among the general population in this border area and more information is required to adapt health policies and intervention programs [11].

The aim of the present study was thus to describe the level of knowledge, attitudes and practices regarding HIV among the populations living on both sides of the border between French Guiana and Brazil.

## Methods

### Study design

**Fig. 1:**
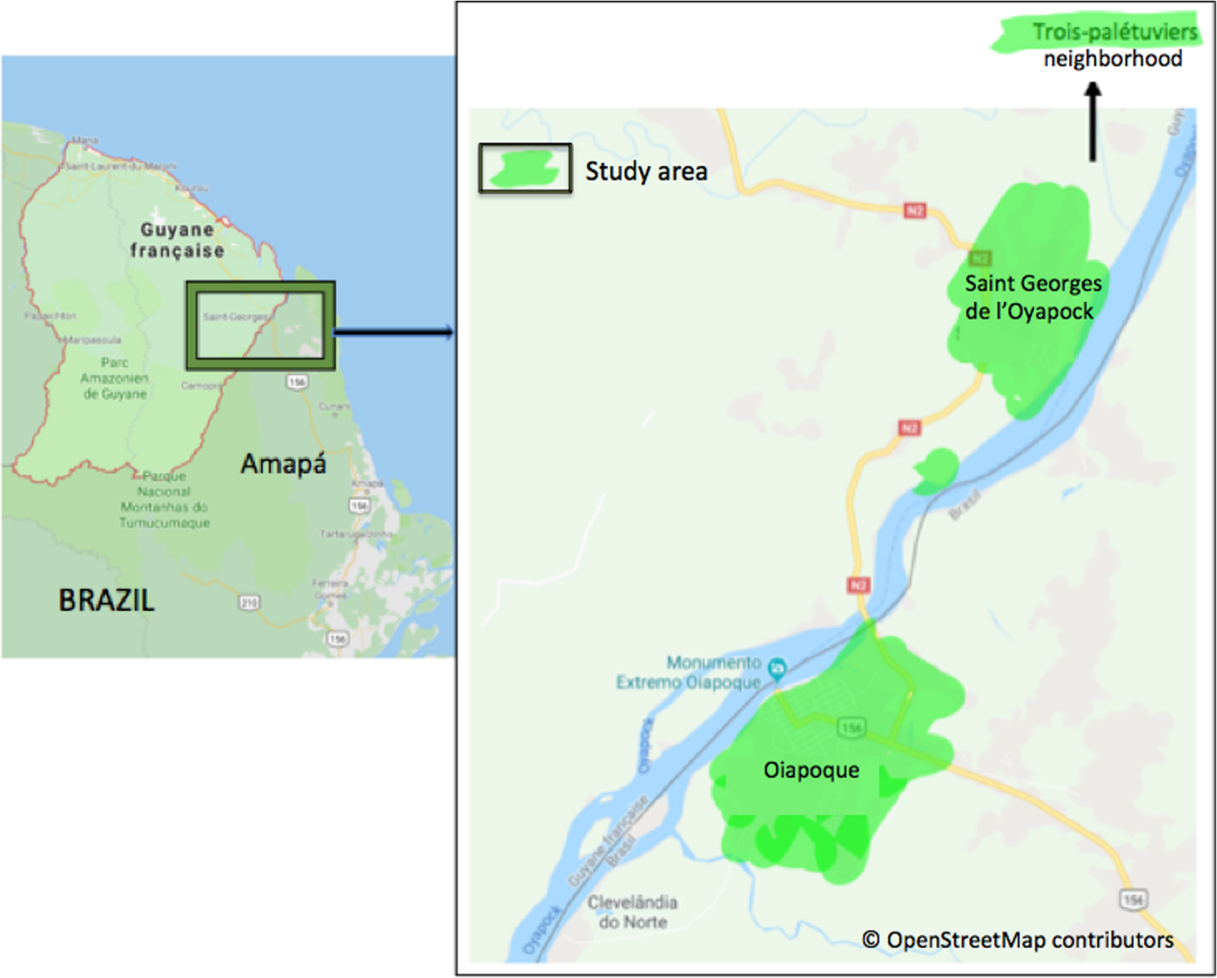
Study area: Saint-Georges-de-l’Oyapock and Oiapoque cities. Source:© OpenStreetMap contributors; shapefile downloaded from https://www.openstreetmap.org and data is available under the Open Database Licence: licensed as CC BY-SA. Created by Emilie Mosnier, 2019

The study was cross-sectional and descriptive. It was a knowledge, attitudes and practices (KAP) study among the inhabitants of the Brazilian border city of Oiapoque and the French Guianese city of Saint-Georges-de-l’Oyapock (STG) (Fig. 1). Data collection took place for 5 months, from October 2017 to February 2018.

This study was an initiative of the “Oyapock Health Cooperation” (OHC) program and was conducted by non-governmental organizations (NGOs):!Dsanté in French Guiana, and DPAC and the Federal Amapá University (UNIFAP) in Brazil [5].

### Sampling method and data collection

All neighborhoods of the two study sites (Oiapoque and STG) were included (Fig. 1). One of every two streets was randomly selected from all the streets in each neighborhood in order to accurately represent all the districts of both border cities. Then, one of either sides of the street was selected. All participants aged 18 years or older in the household who accepted to participate in the study were surveyed.

The questionnaire was created on the basis of a prior HIV knowledge, attitudes and practices (KAP) survey conducted in French Guiana [8, 9]. The data were collected from an anonymous structured questionnaire of 77 questions, in Portuguese or in French. Questions were administered door-to-door in an individual interview setting by trained local interviewers from the local UNIFAP university or from local NGOs: DPAC or!Dsanté. Depending on the translation skills of the interviewers, when needed questions were administered and explained in the participant’s native language (creole, or Amerindian languages) for improved comprehension and minimized information bias.

### Settings and participants

Brazil and French Guiana share a 730 km-long border along the Oyapock River. Traffic and exchange between both countries are concentered in two river border towns: STG (on the French Guiana side) and Oiapoque (on the Brazilian side) (Fig. 1). The average population size in 2017 was approximately 30,000 in Oiapoque and 4500 in STG. Some neighborhoods are only accessible by canoe. Crossing the border is possible by car or canoe and only Brazilians are required to have a visa. The population is multi-ethnic in this border area. We may find Amerindians (mostly Palikur, Galibi, Galibi Marworno and Karipuna communities), French Guianese Creoles, Brazilian immigrants from other States of Brazil and French citizens from other regions outside French Guiana.

### Outcome criteria

This study aimed to investigate three outcome variables: knowledge about HIV, HIV risk behavior and the level of stigmatization.

Poor knowledge about HIV was defined as a “no”, “don’t know” or “don’t want to answer” response from the participant to at least one of the following questions: *Have you ever heard about the disease HIV/AIDS? Can one avoid becoming infected by HIV? Is HIV transmitted through blood? Is HIV transmitted through sperm? Can HIV be transmitted from a pregnant woman to her child during pregnancy? Did you hear or know about HIV treatment?*

High HIV risk behavior (*n* = 127/ 621, 20.5%) was defined as having (i) non-systematic use of condoms with a casual sex partner (*n* = 102, 16,4%), or (ii) commercial sex partners (*n* = 11, 1,8%) and/or (iii) having at least two sexual partners (*n* = 88, 14.0%). Multiple and concurrent partnerships are common in French Guiana and have been previously reported as factors that drive the HIV epidemic [12].

The level of stigmatization was evaluated via 4 questions on the topics of worklife, family and intimate relationships with people living with HIV (PLHIV) (Additional file 1 Data). Two groups were defined, one group presenting no stigmas (0 answers suggesting stigmatizing attitudes) and a second group presenting one or more negative beliefs towards PLHIV (1 to 4 answers suggesting stigmatizing attitudes).

### Statistical analysis

A descriptive analysis was conducted to compare socio-demographic characteristics and sexual behaviors between participants with high or low levels of HIV knowledge, HIV risk behaviors and HIV stigma. Mean and standard deviation for normal distribution variables, and frequencies and percentages for qualitative variables were calculated. Categorical variables were compared between groups using the Pearson’s χ^2^ test or the Fisher’s exact test when at least one of the categorical answers had less than 30 respondents. Comparisons of continuous variables were conducted using the Student’s t-test or AnOVa if there were more than 2 groups to compare and when checked values followed a normal distribution with equal variances.

## Results

Of the 621 study participants, 252 (40.6%) and 369 (59.4%) resided in STG and Oiapoque, respectively.

### Socio-demographic characteristics of the participants (Table 1)

The socio-demographic characteristics of the study participants are presented in Table 1. Females were predominant (M/F sex ratio = 0.65). The mean age was 35.7 IC_95%_ [34.6–36.7] years old. Over one-third of participants (39.5%) were above 30 years of age. The majority of participants (76.9%, *n* = 478/621) indicated Portuguese as their native language, with 49.6% (*n* = 125/252) on the French Guiana side. Most participants on both sides were in a precarious situation with no or little income, and only 16.4% (*n* = 102/621) reported having a paid employment. Table 1 provides a comparison of the participants’ socio-demographic characteristics between the inhabitants of Oiapoque and STG.

**Table 1:** Demographics of survey participants

Characteristics	Inhabitants of Oiapoque (Brazil) Frequency N (row %) *N* = 369	Inhabitants of STG^a^ (French Guiana) Frequency N (row %) *N* = 252	Total Frequency N (col %) *N* = 621	*p* value
Sex				
Male	137 (56.4)	106 (43.6)	243 (39.1)	0.216
Female	232 (61.4)	146 (38.6)	378 (60.9)	
Sexual orientation				
Heterosexual	340 (59.6)	230 (40.4)	571 (92.0)	0.002
Homosexual	13(100.0)	0 (0.0)	13 (2.0)	
Bisexual	5 (71.4)	2 (28.6)	7 (1.2)	
Missing data	10 (33.3)	20 (66.7)	30 (4.8)	
Age				
18–29	138 (56.3)	107 (53.7)	245 (39.5)	0.336
30–44	143 (62.9)	84 (37.0)	227 (36.5)	
≥ 45	88 (59.1)	61 (40.9)	149 (24.0)	
Native language				
French	2 (6.3)	30 (93.8)	32 (5.2)	< 0.001
French Guianese creole	3 (4.8)	60 (95.2)	63 (10.1)	
Portuguese	353 (73.9)	125 (25.2)	478 (77)	
Amerindian	1 (3.1)	31 (96.9)	32 (5.2)	
Others	10 (62.5)	6 (37.5)	16 (2.6)	
Education level				
Any level to middle school	137 (50.6)	134 (49.4)	271 (43.6)	< 0.001
High school to University	232 (66.3)	118 (33.7)	350 (56.4)	
Income				
Paid employment	67 (65.7)	35 (34.3)	102 (16.4)	< 0.001
Informal activity	60 (87)	9 (13)	69 (11.1)	
Social allowance	64 (38.3)	103 (61.7)	167 (26.9)	
None	138 (59.7)	93 (40.3)	231 (37.2)	
Others	37 (80.4)	9 (19.6)	46 (7.4)	
Missing data	3 (50)	3 (50)	6 (1.0)	

^a^Saint-Georges-de-l’Oyapock

### HIV knowledge (Table 2)

Slightly more than half of participants (54.1% *n* = 336/621) showed a high level of HIV knowledge and correctly answered six questions. However, 59.7% (*n* = 371/621) thought or didn’t know if the virus was transmitted by mosquitoes. In addition, 34.6% (*n* = 215/621) thought or didn’t know if the virus was transmitted by sharing a glass with a PLHIV. Only 7.8% (*n* = 49/621) thought or didn’t know that the virus was not transmitted by sperm. When respondents were asked if they believed in protection against the disease through traditional medicine or lucky charms, 14.7% (*n* = 62/523) and 8.3% (*n* = 42/509) responded positively, respectively. The majority of respondents (86.3% *n* = 536/621) did not know about HIV post exposure prophylaxis treatment. However, 78.7% (*n* = 489/621) reported being aware of the existence of HIV treatments for PLHIV. The three best sources of information reported were health caregivers (40.1%, *n* = 362), television (18.3%, *n* = 165) and internet (13.4%, *n* = 121). Table 2 presents the comparison using bivariate analysis of the main characteristics between a low and high level of HIV knowledge.

**Table 2:** Bivariate analysis of participants with good or poor knowledge

Statement (*n* = 621)	Poor HIV knowledge Frequency *N* (row %) *N* = 285	Good HIV knowledge Frequency *N* (row %) *N* = 336	*p* value
Sex n = 621			
Male	121 (49.8)	122 (50.2)	0.103
Female	163 (43.1)	215 (56.9)	
Sexual orientation			
Heterosexual	144 (27.4)	426 (72.6)	0.525
Homosexual	3 (21.4)	11 (78.6)	
Bisexual	1 (14.3)	6 (85.7)	
Missing data	8 (26.6)	22 (73.4)	
Age *n* = 621			
18–29	118 (48.2)	127 (58.8)	0.448
30–44	104 (48.8)	123 (54.2)	
≥ 45	62 (41.6)	87 (58.4)	
Native language			
French or Portuguese	222 (36.4%)	288 (63.6%)	0.011
Creole, Amerindian and others	63 (56.8%)	48 (43.2%)	
Education level			
Any level to middle school	138 (50.9)	133 (49.1)	0.022
High school to University	147 (42.0)	203 (58.0)	
Income			
Paid employment	42 (41.2)	60 (58.8)	0.105
Informal work	35 (50.7)	34 (49.3)	
Social allowance	75 (44.9)	92 (55.1)	
None	100 (43.3)	131 (56.7)	
Others	29 (63.0)	17 (37.0)	
Missing data	3 (50.0)	3 (50.0)	
Mean number of HIV information sources [IC_95%_]	1.65 [1.55–1.76]	2.12 [1.97–2.27]	< 0.001
Last HIV test			
Less than a year	89 (36.5)	155 (63.5)	0.021
More than a year	108 (48.6)	122 (51.4)	
Missing data	87 (59.2)	60 (40.8)	
Would you do an HIV test in the future?			
Yes	230 (43.5)	299 (56.5)	0.066
No	37 (56.1)	29 (43.9)	
Missing Data	17 (65.4)	9 (34.6)	
Do you know a PLHIV^a^?			
Yes	89 (36.5)	155 (63.5)	0.001
No	174 (50.9)	168 (49.1)	
Missing Data	21 (60.0)	14 (40.0)	

^a^PLHIV: people living with HIV

### Factors associated with HIV risk behavior (Table 3)

The median age at which young people had their first sexual relations was 15.9 years IC_95%_ [15.6–16.2]. Men reported earlier sexual activity than women (15.4 vs 16.2 years respectively *p* < 0.001). Over the past year, 17.6% of respondents (*n* = 109) reported having casual sexual partners, in 24.7% (*n* = 60/109) and 13.0% (*n* = 49/109) in men and women respectively (*p* < 0.001). The median number of casual partners was 2.77 IC_95%_ [0.30–5.23]. Men reported a greater number of sexual partners than women (2.8 vs 1.4, *p* < 0.001). A little more than a quarter of participants (28.8% *n* = 30/104) reported high HIV risk behavior with no condom use during their last sexual intercourse with a casual sexual partner (more frequently in women than men *p* = 0.024). Transactional sex was reported only in 1.77% (*n* = 11/621) of cases. Table 3 presents the main characteristics of a bivariate analysis of a high HIV risk behavior group compared with a low HIV risk behavior group.

**Table 3:** Bivariate analysis of high and low HIV risk behavior group

Statement (*n* = 621)	Low HIV risk behavior Frequency *N* (row %) *N* = 494	High HIV risk behavior^a^ Frequency *N* (row %) N = 127	*p* value
Sex			
Male	176 (72.4)	67 (27.6)	0.001
Female	317 (83.9)	61 (16.1)	
Sexual orientation			
Heterosexual	468 (82.1)	102 (17.9)	0.045
Homosexual	8 (57.1)	6 (42.9)	
Bisexual	5 (71.4)	2 (28.6)	
Missing data	12 (40.0)	18 (60.0)	
Mean age at first sexual intercourse [IC_95%_]]	16.03 [15.73–16.34]	15.17 [14.5–15.84]	0.020
Native language			
French	24 (75.0)	8 (25.0)	0.351
French Guianese creole	54 (85.7)	9 (14.3)	
Portuguese	376 (78.7)	102 (21.3)	
Amerindian	24 (75.0)	8 (25.0)	
Others	15 (93.8)	1 (6.2)	
Education level			0.567
Any level to middle school	218 (80.4)	53 (19.6)	
High school to University	275 (78.6)	75 (21.4)	
Income			
Paid employment	78 (76.5)	24 (23.5)	0.012
Informal work	54 (78.3)	15 (21.7)	
Social allowance	147 (88.0)	20 (12.0)	
None	177 (76.6)	54 (23.4)	
Others	32 (69.6)	14 (30.4)	
Missing data	5 (83.3)	1 (16.7)	
Place of residence			
STG^b^ (French Guiana)	209 (82.9)	43 (17.1)	0.071
Oiapoque (Brazil)	284 (774)	84 (223)	
Level of HIV knowledge			
Poor	218 (76.8)	66 (23.2)	0.137
High	275 (81.6)	62 (18.4)	
Prior HIV test *n* = 601			
Yes	394 (82.3)	85 (17.8)	0.012
No	88 (72.1)	34 (27.9)	
Missing Data	11 (55)	9 (45)	

^a^High HIV risk behavior was defined as a non-systematic use of condoms with casual or commercial sex partners or having more than two sexual partners

^b^Saint-Georges-de-l’Oyapock

### Stigmatization against people living with HIV (Table 4)

High levels of HIV stigma were identified: 74.9% (*n* = 465/621) responded with one or more negative beliefs towards PLHIV. Stigmatizing attitudes are greater in situations of close proximity. For example, most participants agree to work with a PLHIV (87.5%, *n* = 525), but only 40.4% (*n* = 251) agree to leave their children with a PLHIV and only 36.2% (*n* = 225) agree to eat a meal prepared by a PLHIV. Stigma was higher on the Brazilian side compared to the French Guianese side (*p* = 0.033), and it was more frequent in participants with a low education level (*p* < 0.001) (Table 4). Creole and Amerindian native language speakers reported a higher level of stigma than others (*p* < 0.001) (Table 4). In addition, the group with stigmatizing attitudes reported less HIV testing uptake in the past (p = 0,004) and rejected the idea of more frequent HIV testing in the future than the other group of participants without stigma (*p* < 0.001) (Table 4).

**Table 4:** Characteristics among participants with or without stigma

Statement (*n* = 621)	No stigma Frequency *N* (row %) *N* = 156	Participants with negative beliefs towards PLHIV* Frequency *N* (row %) *N* = 465	*p* value
Sex			
Male	63 (25.9)	180 (74.1)	0.711
Female	93 (24.6)	285 (75.4)	
Sexual orientation			
Heterosexual	3 (21.4)	11 (78.6)	0.924
Homosexual	144 (25.3)	426 (74.7)	
Bisexual	1 (14.3)	6 (85.7)	
Missing data	8 (26.7)	22 (73.3)	
Age			
18–29	55 (22.4)	190 (77.6)	0.024
30–44	71 (31.3)	156 (68.7)	
≥ 45	30 (20.1)	119 (79.9)	
Native language			
French	11 (34.4)	21 (65.6)	< 0.001
French Guianese creole	5 (7.9)	58 (92.1)	
Portuguese	135 (28.2)	343 (71.8)	
Amerindian	2 (6.2)	30 (93.8)	
Others	3 (18.7)	13 (81.3)	
Education level			
Any level to middle school	49 (18.1)	222 (81.9)	< 0.001
High school to University	107 (30.6)	243 (69.4)	
Income			
Paid employment	36 (35.3)	66 (64.7)	0.013
Informal work	22 (31.9)	47 (68.1)	
Social allowance	30 (18.0)	137 (82.0)	
None	53 (22.9)	178 (77.1)	
Others	13 (28.3)	33 (71.7)	
Missing data	2 (33.3)	4 (66.7)	
City of residence of inhabitants			
STG** (French Guiana)	52 (20.6)	200 (79.4)	0.033
Oiapoque (Brazil)	104 (28.2)	265 (71.8)	
Prior HIV test *n* = 601			
Yes	133 (27.8)	346 (72.2)	0.004
No	23 (18.9)	99 (81.1)	
Ok to do a HIV test in the future *n* = 595			
Yes	146 (27.6)	383 (72.4)	< 0.001
No	10 (15.2)	56 (84.8)	
Level of HIV knowledge			
Poor	67 (23.6)	217 (76.4)	0.420
High	89 (26.4)	248 (73.6)	
HIV risk behaviors			
Low	121 (24.5)	372 (75.5)	0.515
High	35 (27.3)	93 (72.7)	

*The level of stigmatization was evaluated via 4 questions on the topics of worklife, family and intimate relationships with PLHIV. Two groups were defined, one group with no stigma (0 discriminate answers) and a second group with one or more negative beliefs towards PLHIV (1 to 4 discriminate answers)

** STG = Saint-Georges-de l'Oyapock

PLHIV=People living with HIV

## Discussion

### Major findings

This is the first HIV KAP study conducted among the general population in a cross-border area. Overall, HIV knowledge appeared to be worse than in mainstream French Guiana or in “mainland France” general populations. For example, the general population in this area gave more incorrect responses regarding the mode of HIV transmission [13–15]. Of course, the poor knowledge associated with low education levels reflects societal problems in French Guiana and Amapá, which have the lowest results in France and Brazil [16]. Furthermore, specific findings on autochthonous and creole communities speaking Amerindian or creole native languages showed poorer knowledge and greater stigmatizing attitudes than French or Portuguese native language participants. This suggests that this area needs more community information on HIV delivered in native languages by community health workers [17].

The present study showed that 20% (*n* = 127/621) of the surveyed populations had been involved in high risk behavior. Men and younger-aged participants reported more frequent risky sexual behavior. Fewer numbers of reported HIV testing was also associated with risky sexual behavior, suggesting that those engaging in sexual risks were unaware of their high-risk behavior. Furthermore, HIV knowledge was not associated with less HIV risk behavior. These results highlight the difficulty in and the importance of developing a deeper causal relationship between communicating HIV information and decreased high-risk behavior. Nevertheless, HIV testing was associated with good HIV knowledge. Although the causal arrow could point either direction, this is a reassuring factor for prevention campaigns and could participate in reducing undiagnosed infections which drive the epidemic in this area [10, 18].

High levels of stigma toward PLHIV persist along the French Guianese and Brazilian borders, which is comparable to data collected along the Surinam border with French Guiana [9]. Fear of stigma has previously been reported as being associated with a lower use and acceptance of services for HIV testing, care and treatment [19].

Populations on both sides of the border appeared to share similar representations of HIV and could benefit from free HIV testing in both Brazilian or French health care centers [5]. HIV treatment is already available on the French Guiana side of the border and will be available in a few months in the Brazilian city of Oiapoque thanks to bi-national collaborative efforts. This is the building block for comprehensive prevention policies designed to optimize resources from each country, which share precarious and mobile populations in this border area.

### Limitations

This is a declarative study on intimate aspects of one’s life. It is prone to biases, notably underreporting of certain behaviors. The number of non-heterosexual participants and transactional sex in the study was small, possibly underestimated due to the face-to-face questionnaire data collection method, which limits the potential for stratification by gender. No data was collected on drug or alcohol use or degree of wellbeing, which could be associated with risk behaviors.

## Conclusion

These data from Brazilian and French Guianese border general populations represent key first steps in understanding the informational and behavioral context of the HIV epidemic in the specific context of a border area. This information will guide HIV prevention and health policies. HIV knowledge and behaviors appear more related to socio-economic challenges and education level than place of residence. Our study suggests that cooperation and shared cross-border prevention strategies are important. According to our results, targeted community communication for autochthonous and creole populations is necessary.

## Supplementary information

**Additional file 1. Data:** Questions designed to evaluate HIV stigma.

## Data Availability

The datasets generated and analyzed during the current study are not publicly available due to the requirement of special authorization to transfer databases provided by the CNIL. Upon prior *“Commission nationale de l’informatique et des libertés”* (CNIL) authorization, the datasets can be made available from the corresponding author upon reasonable request.

## Abbreviations

CNIL: Commission Nationale Informatique et Libertés
HIV: Human Immunodeficiency virus
KAP: Knowledge, Attitudes, Behaviors and Practices
NGO: Non-Governmental Organization
OHC: Oyapock Health Cooperation program
PLHIV: People living with HIV
PrEP: Pre-exposure prophylaxis
STG: Saint-Georges-de-l’Oyapock
UNIFAP: Federal Amapá University of Brazil
