# Supplementary material for: Knowledge, attitudes, practices about HIV and implications in risk and stigma prevention among French Guianese and Brazilian border inhabitants: Beliefs about HIV among border inhabitants
Source: BMC Public Health. 2019 Dec 4;19:1633. doi: 10.1186/s12889-019-7997-1 (PMC6894142; doi:10.1186/s12889-019-7997-1)
Supplement: Supplementary file 1 — Additional file 1. Data: Questions designed to evaluate HIV stigma. [file 12889_2019_7997_MOESM1_ESM.docx]

**Supplementary data:**

**Supplementary data 1:** Questions designed to evaluate HIV stigma:

1. If you know someone who is HIV-infected, would you agree to work with that person?
2. If you know someone who is HIV-infected, would you agree to eat with that person?
3. If you know someone who is HIV-infected, would you agree to let your child be kept with that person?
4. If you know someone who is HIV-infected, would you agree to hire that person to cook?
